# Video mirror feedback induces more extensive brain activation compared to the mirror box: an fNIRS study in healthy adults

**DOI:** 10.1186/s12984-024-01374-1

**Published:** 2024-05-14

**Authors:** Julien Bonnal, Canan Ozsancak, Fabrice Prieur, Pascal Auzou

**Affiliations:** 1 Service de Neurologie, Centre Hospitalier Universitaire d’Orléans, 14 Avenue de l’Hôpital, Orleans, 45100 France; 2 CIAMS, Université Paris-Saclay, Orsay Cedex, 91405 France; 3 CIAMS, Université d’Orléans, Orléans, 45067 France; 4 SAPRéM, Université d’Orléans, Orléans, France; 5 LI2RSO, Université d’Orléans, Orléans, France

**Keywords:** Mirror therapy, Virtual reality, Precuneus, Motor cortex, Cerebral activation, fNIRS

## Abstract

**Background:**

Mirror therapy (MT) has been shown to be effective for motor recovery of the upper limb after a stroke. The cerebral mechanisms of mirror therapy involve the precuneus, premotor cortex and primary motor cortex. Activation of the precuneus could be a marker of this effectiveness. MT has some limitations and video therapy (VT) tools are being developed to optimise MT. While the clinical superiority of these new tools remains to be demonstrated, comparing the cerebral mechanisms of these different modalities will provide a better understanding of the related neuroplasticity mechanisms.

**Methods:**

Thirty-three right-handed healthy individuals were included in this study. Participants were equipped with a near-infrared spectroscopy headset covering the precuneus, the premotor cortex and the primary motor cortex of each hemisphere. Each participant performed 3 tasks: a MT task (right hand movement and left visual feedback), a VT task (left visual feedback only) and a control task (right hand movement only). Perception of illusion was rated for MT and VT by asking participants to rate the intensity using a visual analogue scale. The aim of this study was to compare brain activation during MT and VT. We also evaluated the correlation between the precuneus activation and the illusion quality of the visual mirrored feedback.

**Results:**

We found a greater activation of the precuneus contralateral to the visual feedback during VT than during MT. We also showed that activation of primary motor cortex and premotor cortex contralateral to visual feedback was more extensive in VT than in MT. Illusion perception was not correlated with precuneus activation.

**Conclusion:**

VT led to greater activation of a parieto-frontal network than MT. This could result from a greater focus on visual feedback and a reduction in interhemispheric inhibition in VT because of the absence of an associated motor task. These results suggest that VT could promote neuroplasticity mechanisms in people with brain lesions more efficiently than MT.

**Clinical trial registration:**

NCT04738851.

## Introduction

Mirror therapy (MT) is commonly used for stroke rehabilitation. This technique consists of using the reflection in a mirror of the movements of a healthy limb to give the illusion of movement of the pathological limb. First proposed for phantom limb pain [1], MT was then used for motor rehabilitation of the post-stroke hemiparetic upper limb [2]. Recent meta-analyses have reported a beneficial effect of MT on upper limb motor recovery after stroke [3, 4].

Despite its effectiveness, the use of MT may be limited by difficulty with positioning for individuals with postural deficits, the need for bilateral training, or associated disorders such as aphasia or hemispatial neglect [5, 6]. New MT tools using virtual reality have been developed to improve the technique [7]. In this study, we focused on video therapy (VT) in which the mirror is replaced by a digital screen [8–10]. The use of these recent tools has been found to be feasible [11]. To our knowledge, there is no evidence of clinical superiority of VT over MT. The relatively high cost of these technologies makes it necessary to determine if they are indeed more effective than simpler, lower cost tools [7]. As such, it seems relevant to compare brain activation patterns between both modalities (MT and VT).

Many studies have explored the brain mechanisms of MT in both people after stroke and healthy individuals. MT activates the motor cortex, in particular the primary motor cortex (M1), premotor cortex (PMC) [12–15] and the precuneus (PC) [16–19] contralaterally to the side of visual feedback. In this study we focused more specifically on the activation of the PC as a determining factor of the effectiveness of the technique. Indeed, it has been shown that motor recovery following MT is correlated with PC activation [19]. One of the roles of the PC is to integrate the visual information from the environment and its transmission to the motor cortex to create a body self-perception [20]. Therefore, in MT the PC could be activated when the visual feedback gives the illusion of ownership of the visualized limb. It then seems relevant to assess the correlation between this activation and the quality of perception of the illusion.

Among the studies evaluating brain activity during MT, some used a real mirror [12, 13, 15, 17] and others a VT tool [14, 16, 18, 19], often for reasons of compatibility with the imaging method. To our knowledge, no study has directly compared the brain activation profiles of these 2 techniques. It seems appropriate to study these mechanisms in healthy subjects as a first step, in order to provide a rationale for future studies in patients. The literature on MT has shown similar activation patterns between healthy subjects [13, 16] and stroke subjects [14, 19]. A MT study conducted in healthy and stroke subjects found precuneus activation in both populations [21].

We chose to use fNIRS to determine the amount of activation of the cerebral regions of interest. This technique enables the evaluation of neurovascular coupling by measuring changes in both oxyhemoglobin (HbO_2_) and deoxyhemoglobin (HbR) in the cortex. The portability of the fNIRS device means it can be used in the real-life environment, including to determine the cerebral mechanisms involved in rehabilitation [12, 13, 16, 19].

The first aim of the study was to compare cerebral activation (PC, PMC and M1) induced by MT and VT tasks using functional near infrared spectroscopy (fNIRS). We hypothesized that VT would lead to greater activation of each region. The second aim of this study was to evaluate the correlation between individuals’ perceptions of the illusion of movement for the two mirrored feedback modalities (MT and VT) and brain activation. We hypothesised that the stronger the illusion of movement, the greater the activation of the PC.

## Materials and methods

### Participants and ethical statement

Thirty-three right-handed individuals (9 males, 24 females; mean (SD) age 24.5 (3.4) years, range 19–40) with no history of neurological, physical, or psychiatric illness were included in this study. Two other individuals were initially recruited, but their data could not be analysed owing to the poor quality of the fNIRS signal. The Edinburgh Handedness Inventory [22] was used to evaluate handedness. All participants had an Edinburgh laterality ratio ≥ 80. Full written consent was obtained from all participants in accordance with the Declaration of Helsinki. This study was approved by the Institutional Review Board CPP NORD-OUEST I on 21st January 2021 (no. 2020-A02936-33) and was registered on clinical trials.gov (NCT04738851). The study is reported according to the Strengthening the Reporting of Observational Studies in Epidemiology (STROBE) guidelines.

### Experimental design and procedure

**Fig. 1:**
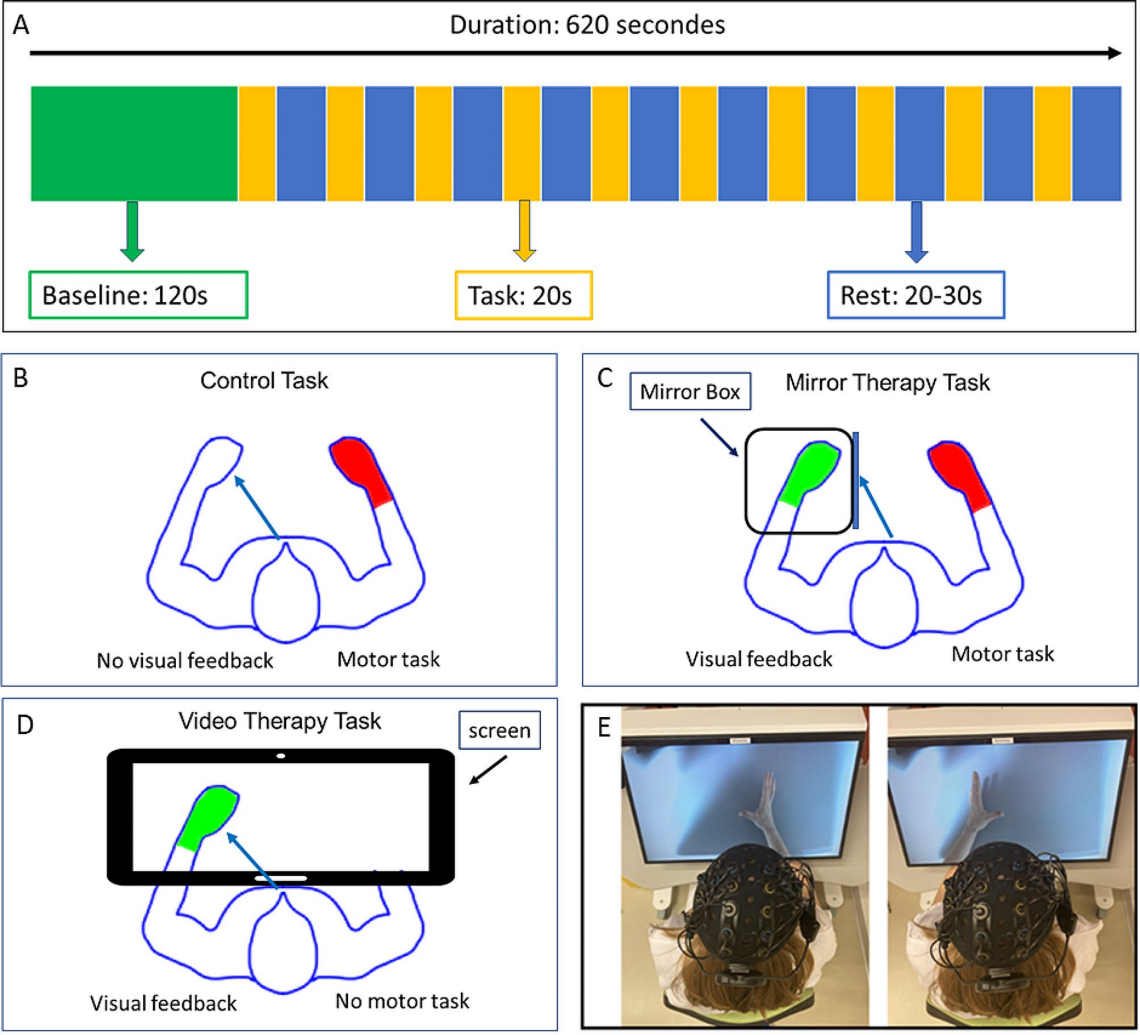
experimental design. (A) Block design for each recording. (B) Control Task. (C) Mirror Therapy Task. (D) Video Therapy Task. The blue arrow represents the direction of the participant’s gaze. The green hand indicates the provision of mirrored feedback and the red hand indicates that the participant is performing a motor task. (E) Picture of the setup of the Video Therapy Task: on the left, the movement recording with the right hand and on the right the visualization of the movement on the left after flipping the image

Participants were required to sit in a comfortable, upright position during the experiment. Experiments were organized in a block paradigm (Fig. 1A). The block design included 10 20-s trials for each task. Rest time between trials varied from 20 to 30 s to minimize the physiological effects of respiration, heart rate, and Mayer waves (low-frequency oscillations in blood pressure) on hemodynamic responses to the task [23].

Each participant completed 3 separate recordings (with a 10-minute rest period in between) for 3 different tasks. The control task involved performing a movement with the right hand while looking at the left hand (Fig. 1B). During this task, the left hand is motionless. So, this task has been carried out to check that the results related to the mirror techniques are not only attributable to the observation of the hand (in this case, the left hand) but require the visualization of a movement. The mirror therapy task involved performing a movement with the right hand while observing the reflection of that movement in a mirror (Fig. 1C). For this task, a mirror box was used in which the left hand was positioned. The video therapy task involved observing a left-hand movement on a screen (with the left hand placed under the screen) (Fig. 1D). During VT task, no movement was performed by the participant’s right hand. For VT task, we used an innovative device based on the principle of mirror therapy and action observation using a screen instead of a normal mirror. We used the IVS3 (Dessintey®, Saint-Etienne, France). This tool requires the pre-recording of a movement with one hand (the healthy hand in the case of a stroke), which is then flipped into the contralateral hand (the impaired hand in the case of a stroke). For this study, movement of the participant’s right hand was recorded before the performance of the task and then the illusion of movement of the left hand was provided on the screen (Fig. 1E). For each task, the movement studied was a hand opening/closing movement performed at a frequency of 0.5 Hz (using a metronome) [24]. The order of the 3 conditions was counterbalanced to avoid any effect of the order. The perception of the illusion was rated for each of the mirror feedback tasks (MT and VT) by asking participants to rate the intensity of any sensations they experienced (tingling, warmth, desire to move the hand, sensation of contraction, etc.) using a visual analogue scale.

### fNIRS data acquisition

**Fig. 2:**
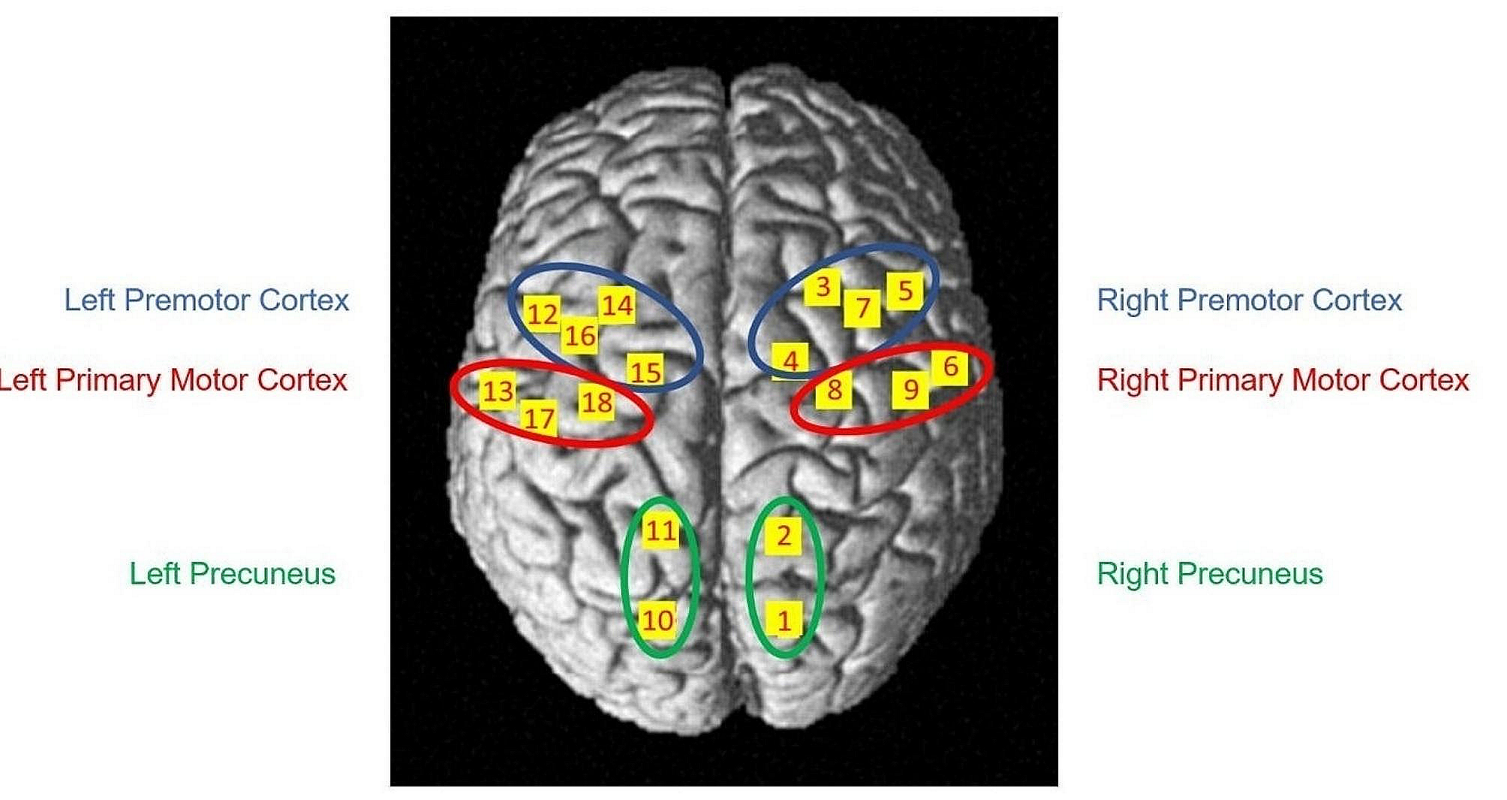
Anatomical locations of the channels and representation of the Regions of Interest superimposed onto the normalized brain surface in the MNI standard brain template

Changes in the concentrations of oxyhemoglobin (HbO_2_) and deoxyhemoglobin (HbR) within the cerebral cortex were measured using a continuous wave optical system Brite 24 system (Artinis Medical Systems, Netherlands). The sources of this system generate 2 wavelengths of near-infrared light at 670 and 850 nm, and the sampling rate is fixed at 10 Hz. A total of 10 light sources and 8 detectors with an inter-optode distance of 3 cm constituted 18 channels (Fig. 2).

To localize the coordinates of each channel in the Montreal Neurological Institute standard brain [25], a 3D digitizer (FASTRACK, Polhemus) was used, and the coordinates were further imported to the NIRS SPM toolbox for spatial registration [26]. The coordinates were then used to define the channels constituting the different regions of interest (ROI) that were used for the statistical analysis (Fig. 2). We defined 3 ROI for each hemisphere as follows: Right PC (Channels 1,2), Right M1 (channels 6,8,9), Right PMC (Channels 3,4,5,7), Left PC (Channels 10,11), Left M1 (channels 13,17,18) and Left PMC (channels 12,14,15,16).

### Preprocessing of fNIRS data

We used both HbO_2_ and HbR signals to measure the hemodynamic response because they provide different and complementary information [27, 28]. The Homer2 toolbox in Matlab (The MathWorks Inc.) was used for offline data preprocessing [29].

The processing was performed as follows:

1. Identification and exclusion of bad channels: channels were considered as bad and excluded from the analysis if the coefficient of variation ([standard deviation/mean]*100) of the raw data was > 33%. The function hmrPruneChannels was used (SNRthresh = 3). For each subject, the number of channels excluded ranged from 0 to 6. Overall, 5% of channels were excluded from the analysis.
Optical density conversion: raw data were converted into optical density with the hmrIntensity2OD function.
Filtering periodic noise: respiration, cardiac activity and high frequency noise were attenuated with hmrBandpassFilt (hpf = 0, lpf = 0.1).
For the remaining artifacts (physiological and motion artifacts), Principal Component Analysis was used with the enPCAfilter_nSV function.
Concentration conversion: corrected optical density data were converted into relative concentration changes with the modified Beer-Lambert law [30]. The age-dependent differential path length factor (DPF) value was calculated for each participant [31]. DPF values were calculated for each wavelength according to the mean age. They were respectively 6.2 and 5.1 for the 760 and 840 nm wavelengths.
The hemodynamic response function was estimated by solving a general linear deconvolution model using the hmrDeconvTB_SS3rd function (t range = [-5, 25], gstd = 1, gms = 1, rhoSD_ssThresh = 1).

### Data analysis

Data analysis was performed with Matlab (The MathWorks Inc.). Mean values were calculated for the rest (from 5 s before, to the beginning of the task) and trial periods (from + 5 s to + 25 s) for each channel. To detect cerebral activation, the mean changes in HbO_2_ and HbR between the rest period and task for each channel and for each ROI were compared using the unilateral paired Student t test. For the ROI analysis, an average of the corresponding channels was made. We applied a Benjamini–Hochberg procedure [32] to control the growth of the false discovery rate (FDR) caused by multiple comparisons. The task comparisons were analysed using one-way repeated measures ANOVA (factor task) for each ROI and HbO_2_, which seems to be a better marker of cerebral activation than HbR [28, 33]. A post-hoc analysis was performed using unilateral paired t-tests. Significance was set at *p* < 0.05 (Bonferroni correction *p* < 0.017).

Finally, we evaluated the link between movement illusion during mirrored feedback (for MT and VT tasks) and PC activation using a Pearson correlation between the Visual Analog Scale and the mean HbO_2_ changes for the Right PC ROI.

## Results

### Comparison of baseline and task hemodynamic responses: cerebral activation

**Fig. 3:**
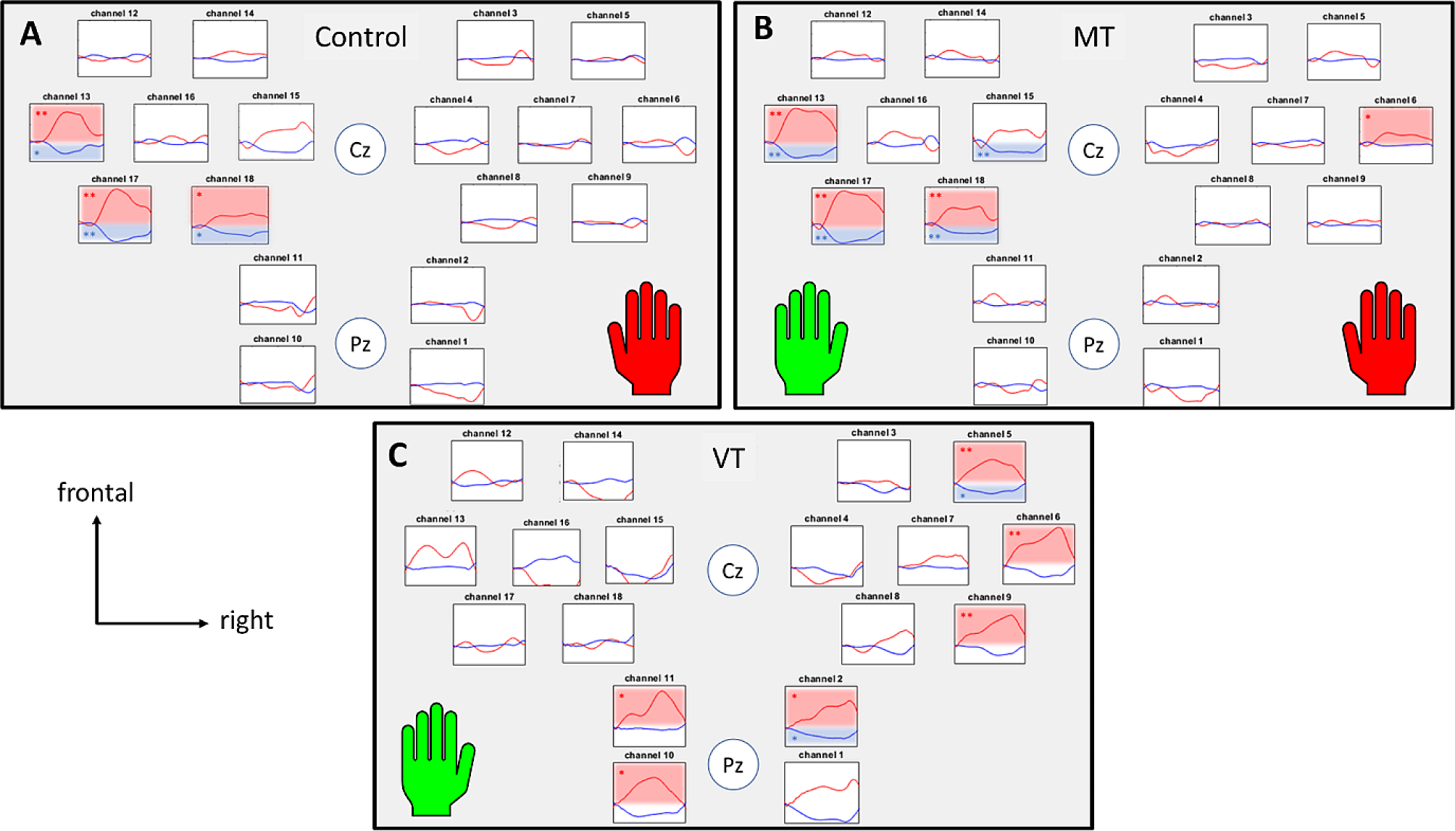
Results of the hemodynamic response by task for each channel. (A) Control Task. (B) Mirror Therapy Task. (C) Video Therapy Task. Results are expressed as means (average of the participants’ HbR and HbO_2_ concentrations). The green left hand indicates that the task involved mirrored feedback and the red right hand indicates that the task involved motor execution. Graph locations were organised according to the anatomical correspondence using the EEG 10/20 system. The time window analysed was 30 s: from 5 s before the beginning of the task to 25 s after. The red traces indicate HbO_2_ concentrations and the blue traces indicate HbR concentrations. The red boxes indicate a significant difference between rest and task periods for HbO_2_ concentration. The blue boxes indicate a significant difference between rest and task periods for HbR concentrations. *: *p* < 0.05; **: *p* < 0.01; FDR corrected

**Fig. 4:**
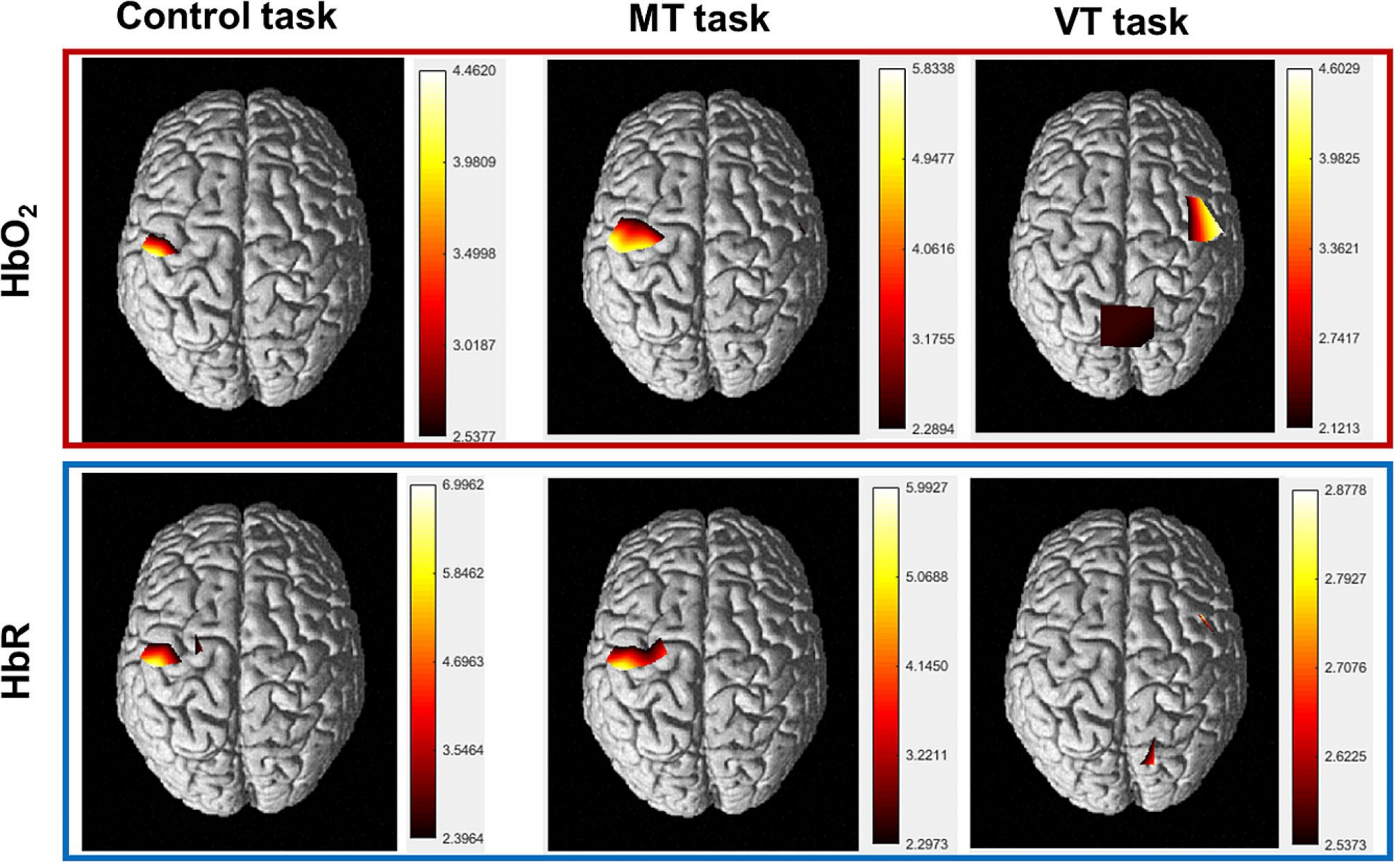
Mean cerebral cortex activation maps for oxyhemoglobin and deoxyhemoglobin during the 3 tasks. Data are *t* values, *t*: statistical value of sample *t*-test with a significance level of *p* < 0.05 (FDR corrected). The change from red to yellow indicates that the degree of activation is from low to high. Only statistically significant responses are illustrated. The data and maps were calculated and generated by NIRS-SPM.

The hemodynamic responses for the 3 conditions (Table 1) are illustrated by the plotogramms (Fig. 3) and a NIRS-SPM (statistical parametric mapping for near-infrared spectroscopy) representation (Fig. 4). Overall, responses were canonical with an increase in HbO_2_ concentration and a tendency towards a decrease in HbR concentration.

**Table 1:** Changes in HbO_2_ and HbR concentrations for ROI and individual channels, and the corresponding *p*-values between rest and task for each condition

	Control Task				Mirror Therapy Task				Video Therapy Task			
	HbO_2_	*p* value	HbR	*p* value	HbO_2_	*p* value	HbR	*p* value	HbO_2_	*p* value	HbR	*p* value
Right PC	-0.066(0.163)	0.991	0.001(0.068)	0.598	-0.039(0.183)	0.883	-0.003(0.068)	0.392	0.043(0.096)	0.007*	-0.031(0.063)	0.004*
Channel 1	-0.111(0.184)	0.999	0.004(0.081)	0.610	-0.083(0.218)	0.982	-0.004(0.084)	0.387	0.0414(0.114)	0.024	-0.039(0.082)	0.005*
Channel 2	-0.027(0.159)	0.834	0.001(0.067)	0.509	0.005(0.181)	0.436	-0.002(0.063)	0.417	0.046(0.115)	0.015*	-0.024(0.059)	0.014
Right PMC	-0.032(0.112)	0.904	0.005(0.060)	0.674	-0.011(0.134)	0.796	0.006(0.074)	0.545	0.019(0.067)	0.258	-0.014(0.049)	0.136
Channel 3	-0.046(0.120)	0.982	0.015(0.058)	0.920	-0.041(0.162)	0.913	0.021(0.090)	0.902	0.004(0.101)	0.404	-0.015(0.066)	0.097
Channel 4	-0.072(0.247)	0.935	-0.008(0.117)	0.350	-0.070(0.245)	0.931	-0.023(0.176)	0.231	-0.030(0.132)	0.885	-0.010(0.102)	0.291
Channel 5	0.012(0.121)	0.280	0.015(0.073)	0.866	0.049(0.157)	0.037	0.004(0.075)	0.607	0.055(0.084)	< 0.001**	-0.024(0.046)	0.004*
Channel 7	-0.024(0.117)	0.868	0.001(0.067)	0.508	-0.016(0.159)	0.711	0.015(0.065)	0.895	0.027(0.088)	0.047	-0.002(0.047)	0.407
Right M1	0.008(0.118)	0.397	-0.006(0.073)	0.352	0.029(0.138)	0.078	-0.008(0.063)	0.298	0.047(0.082)	< 0.001**	-0.016(0.058)	0.026
Channel 6	0.041(0.138)	0.049	-0.014(0.084)	0.170	0.075(0.172)	0.008*	-0.012(0.067)	0.138	0.078(0.096)	< 0.001**	-0.023(0.057)	0.014
Channel 8	-0.023(0.174)	0.774	0.004(0.071)	0.633	0.002(0.178)	0.469	0.004(0.065)	0.639	0.011(0.105)	0.270	-0.009(0.057)	0.175
Channel 9	0.003(0.124)	0.449	-0.008(0.087)	0.293	0.014(0.154)	0.289	-0.014(0.099)	0.212	0.060(0.095)	< 0.001**	-0.018(0.072)	0.081
Left PC	-0.048(0.172)	0.893	-0.002(0.072)	0.422	-0.019(0.185)	0.595	-0.015(0.092)	0.253	0.060(0.134)	0.010*	-0.013(0.073)	0.117
Channel 10	-0.077(0.184)	0.987	0.007(0.073)	0.694	-0.037(0.194)	0.850	-0.003(0.097)	0.425	0.059(0.144)	0.015*	-0.027(0.079)	0.034
Channel 11	-0.013(0.193)	0.649	-0.012(0.087)	0.219	0.009(0.214)	0.404	-0.023(0.105)	0.111	0.065(0.158)	0.015*	-0.004(0.093)	0.417
Left PMC	0.052(0.140)	0.188	-0.011(0.123)	0.258	0.054(0.146)	0.049	-0.035(0.091)	0.066	-0.026(0.107)	0.956	0.001(0.040)	0.265
Channel 12	0.033(0.153)	0.109	0.029(0.129)	0.894	0.035(0.167)	0.129	-0.010(0.089)	0.267	0.009(0.154)	0.378	-0.003(0.037)	0.366
Channel 14	0.012(0.106)	0.256	-0.012(0.111)	0.268	0.020(0.192)	0.279	-0.019(0.120)	0.185	-0.047(0.118)	0.981	0.002(0.033)	0.634
Channel 15	0.077(0.244)	0.057	-0.057(0.147)	0.027	0.077(0.204)	0.027	-0.089(0.114)	< 0.001**	-0.043(0.101)	0.982	-0.030(0.078)	0.032
Channel 16	0.033(0.142)	0.095	-0.007(0.168)	0.394	0.064(0.196)	0.037	-0.015(0.099)	0.196	-0.055(0.178)	0.949	0.027(0.081)	0.961
Left M1	0.151(0.157)	< 0.001**	-0.061(0.090)	< 0.001**	0.204(0.168)	< 0.001*	-0.110(0.123)	< 0.001**	0.015(0.101)	0.208	0.004(0.061)	0.684
Channel 13	0.174(0.183)	< 0.001**	-0.045(0.099)	0.006*	0.244(0.238)	< 0.001*	-0.111(0.161)	< 0.001**	0.045(0.130)	0.030	0.002(0.062)	0.591
Channel 17	0.201(0.212)	< 0.001**	-0.092(0.108)	< 0.001**	0.243(0.249)	< 0.001*	-0.132(0.123)	< 0.001**	-0.003(0.156)	0.555	0.002(0.075)	0.567
Channel 18	0.071(0.130)	0.002*	-0.042(0.092)	0.006*	0.125(0.148)	< 0.001*	-0.071(0.119)	< 0.001**	0.003(0.085)	0.411	0.008(0.064)	0.762

Data are mean(SEM), unit: µMol/L.

Abbreviations: HbO_2_ = oxyhemoglobin; HbR = deoxyhemoglobin; PC = Precuneus; PMC = Premotor Cortex; M1 = Primary Motor Cortex

Significance levels were corrected for multiple comparisons using the Benjamini–Hochberg procedure: * *p* < 0.05; ** *p* < 0.01

#### Control Task

HbO_2_ concentration increased (t = 5, *p* < 0.01) and HbR concentration decreased (t = -3.65, *p* < 0.01) only in the left M1. No significant changes were found for the other ROI or channels.

#### Mirror therapy task

HbO_2_ concentration increased (t = 6.37, *p* < 0.01) and HbR concentration decreased (t = -5.29, *p* < 0.01) in the left M1. No significant changes were found for the other ROI, but Channel 15 (part of left PMC) showed a significant decrease in HbR concentration (t = -3.95, *p* < 0.01) and channel 6 (part of right M1) showed a significant increase in HbO_2_ concentration (t = 2.55, *p* < 0.05).

#### Video therapy task

HbO_2_ concentration increased (t = 2.58, *p* < 0.05) and HbR concentration decreased (t = -2.85, *p* < 0.05) in the right PC. HbO_2_ concentration also increased in the left PC (t = 2.43, *p* < 0.05) and the right M1 (t = 4.19, *p* < 0.01). No significant changes were found for the other ROI, but Channel 5 (part of the right PMC) showed an increase in HbO_2_ concentration (t = 3.72, *p* < 0.01) and a decrease in HbR concentration (t = -2.88, *p* < 0.05).

### Task comparisons

**Fig. 5:**
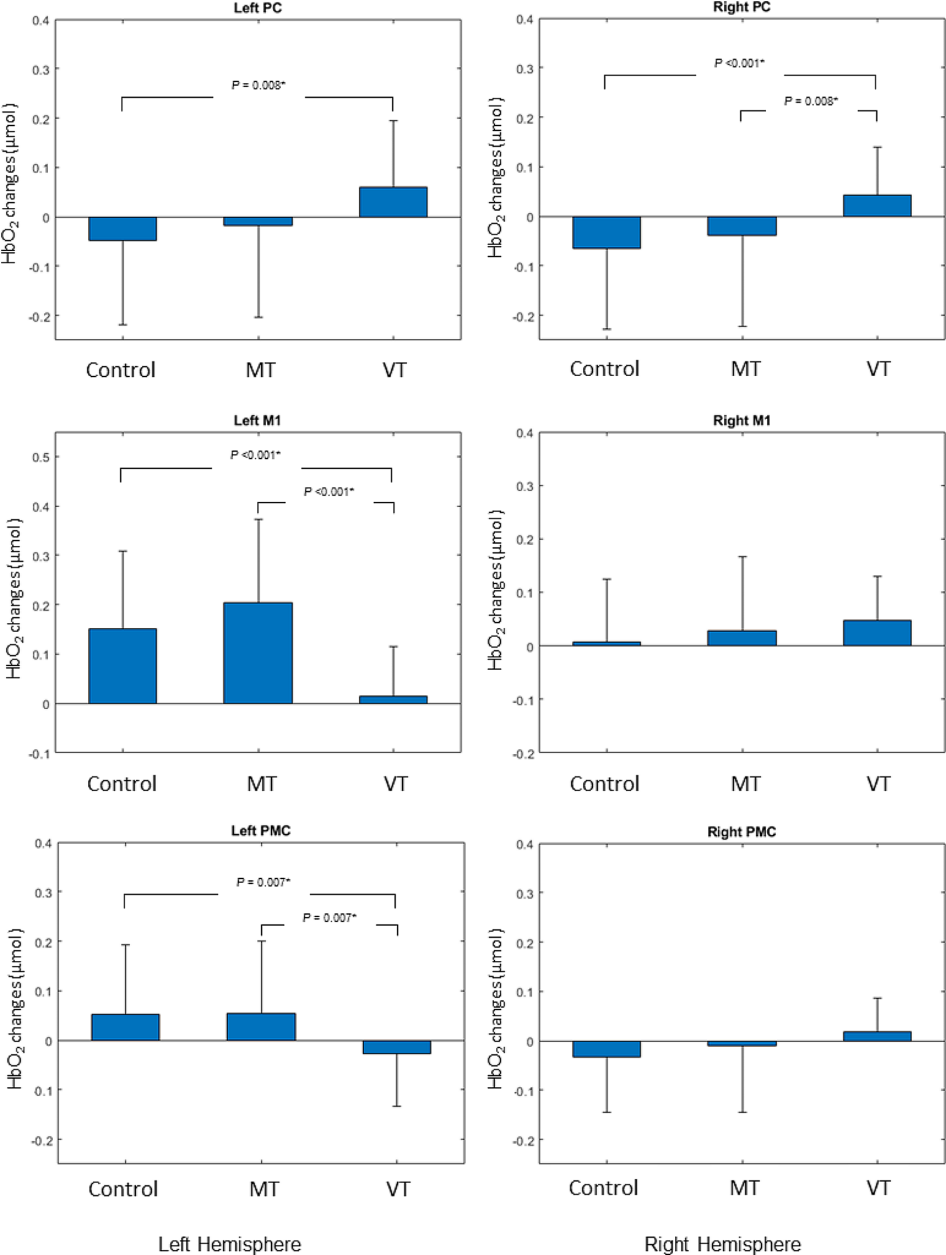
Post-hoc analysis with the paired t-test results that remained significant after Bonferroni correction

The results of the ANOVA and the post-hoc analysis are shown in Fig. 5.

#### Precuneus

For the right PC, one-way ANOVA showed a significant effect of task (F = 5.36, *p* = 0.007). Post-hoc analysis showed that activation was greater during VT task than MT task (t = 2.57, *p* = 0.008) and control task (t = 4.38, *p* < 0.001).

For the left PC, the one-way ANOVA showed a significant effect of task (F = 4.17, *p* = 0.02). Post-hoc analysis showed that activation was greater during VT task than control task (t = 2.56, *p* = 0.008).

#### Primary motor cortex

The one-way ANOVA showed a significant effect of task only for the left M1 (F = 22.32, *p* < 0.001). Post-hoc analysis showed that activation was greater during MT task than VT task (t = 5.7, *p* < 0.001) and during control task than VT task (t = 3.74, *p* < 0.001).

There was no significant effect on the right M1 (F = 1.17, *p* = 0.32).

#### Premotor Cortex

The one-way ANOVA showed a significant effect of task only for the left PMC (F = 7,32, *p* = 0.001). Post-hoc analysis showed that activation was greater during MT task than VT task (t = 2.65, *p* = 0.007) and during control task than VT task (t = 2.67, *p* = 0.007).

There was no significant effect on the right PMC (F = 2.44, *p* = 0.1).

### Illusion of movement

Illusion of movement was evaluated after MT task (mean (SD) 4.5 (2.4)) and VT task (mean (SD) 5.2 (2.2)). There was no correlation between perception of illusion and changes in HbO_2_ for the right PC area of interest for MT task (r² = 0.03, *p* = 0.76) or VT task (r² = 0.07, *p* = 0.14).

## Discussion

To our knowledge, this study is the first to compare the cerebral activation induced by conventional mirror therapy (MT) with that induced by video therapy (VT). In VT, the visualised movements are pre-recorded and projected onto a large screen positioned in front of the individual. Compared with conventional MT, VT could provide a higher quality illusion and encourage attention to visual feedback. This new modality could therefore improve the effectiveness of MT by optimising the mechanisms that induce neuroplasticity. In this study we focused on the regions of interest that are involved in MT, the precuneus (PC), primary motor cortex (M1) and premotor cortex (PMC). We explored mirror tasks with no movement intention, which allowed us to specifically assess the effect of visual feedback. Our main aim was to evaluate the difference in activation of the PC between the two techniques since PC activation could be correlated with the effectiveness of the technique [19]. As we had hypothesized, we found greater activation of the PC contralateral to visual feedback during VT than during conventional MT. We also showed that activation of M1 and PMC contralateral to visual feedback was more extensive in VT than in MT.

### Activation of the PC: movement illusion or attentional mechanisms?

The involvement of the PC in MT has been previously widely demonstrated [16, 17, 19, 34]. The PC plays a major role in visual processing and self-perception [20], and more particularly in the perception of the hand [35]. We initially hypothesized that during the MT and the VT tasks, the PC would be activated if the participants perceived the illusion of seeing their own hand during the visual feedback situation, and we hypothesised that the quality of the illusion would be greater with VT. To verify our hypothesis, we assessed the participants’ perceptions of the quality of the illusion (impression that the hand was moving, tingling, sensations of contraction, etc.), as suggested by Rossiter [36]. We found that the perception of the illusion did not differ between the VT and MT tasks and that it was not correlated with the level of PC activation. Illusion perception is subjective and difficult to assess, in particular because no validated scales exist for that purpose. However, some studies have found PC activation during VT without a real illusion [16, 19]. In those studies, the screen was located at a distance from the participant that did not allow visual continuity between the upper limb and the visual feedback. Thus, we cannot conclude that the greater PC activation found in this study with VT than MT was the result of a higher quality movement illusion with VT.

Another explanation for these results could relate to the role of the PC in attentional tasks. When a person is focused on a given task, the PC may be recruited to support the cognitive engagement. A functional MRI (fMRI) study on 10 healthy individuals showed that the PC was activated during a visuospatial attention task [37]. In addition, a meta-analysis showed that PC lesions could cause spatial hemineglect [38]. The PC therefore seems to be particularly involved in visual attention processes. In our study, the lack of an associated motor task during the VT task may have focused attention on the visual feedback to a greater extent than the MT task. Thus, the more extensive PC activation during VT than MT may have been due to a higher level of attention.

Moreover, in our study PC activation was not only contralateral to the side of feedback but bilateral. This could be explained by the fact that the function of the PC is not as lateralized as that of other brain structures. Indeed, although several MT studies have found that PC activation was strictly contralateral in response to visual feedback [16, 18, 19], two motor imaging studies reported that lateralization of the PC was random across individuals. This result is interesting insofar as in the event of a hemispheric lesion including the PC, its activity could be compensated for by the contralesional PC.

The activity of the PC seems important for rehabilitation, as it could have a predominant role in the stimulation of neuroplasticity. Indeed, it has been shown that the PC is closely connected to the motor cortex [39]. The motor cortex is often damaged after a stroke in individuals with residual upper limb impairment. Activating the PC during rehabilitation could therefore stimulate the ipsilesional M1. Based on these results, it is possible that VT, by improving recruitment of precunei, is an effective technique for improving neuroplasticity and therefore motor recovery in stroke patients. These hypotheses will need to be verified in future clinical studies.

### Other cortical regions of interest: M1 and PMC

First, we only found activation of the left M1 during the MT and control tasks, i.e., the tasks that required motor activity of the right hand. These results are in line with the classical literature regarding the contralateral cortical control of motor activity [40].

Second, we found activation of the right M1 for the tasks involving mirror feedback (MT and VT). These results are also in agreement with the existing literature [12, 13]. However, although we didn’t find any statistical difference in the task comparison for this region, our results show a more extensive activation during VT task than during MT task (for this ROI, one channel was activated during MT task and two channels during VT task). We previously argued that the lack of a motor task during the VT is interesting because it encourages attention on the visual feedback. This absence of a motor task could also explain the difference in M1 activation between VT and MT for two reasons. First, the VT used here, due to the absence of a motor task, could be considered as action observation (AO) therapy associated with visual illusion. A study has shown that AO regulates interhemispheric interaction, with a facilitatory effect on the M1 contralateral to the observed movement and an inhibitory effect on the M1 ipsilateral [41]. However, the VT used here also provided the visual illusion of movement of the own limb. One study showed that AO was able to induce neuroplasticity on M1 only if associated with an illusion of movement (kinaesthetic in the study) [42]. Therefore, the VT used here could have a facilitatory effect on the ipsilesional side (in the case of a stroke) and stimulate neuroplasticity in the M1. Second, the less extensive activation during MT may result from interhemispheric inhibition induced by the right-hand motor task. Indeed, it has been shown that unilateral movement leads to inhibition of the ipsilateral hemisphere via the transcallosal pathway [43]. Interhemispheric balance is altered by stroke [44], even in a resting situation [45], and MT in particular helps to restore this balance [46]. Therefore, it is likely that the difference found in this study would be more marked in a group of people after stroke. However, these results need to be interpreted with caution. Indeed, even though activation involves more channels in VT and is consequently more extensive, our results show no statistically significant difference between the two techniques for the whole ROI. It has also been shown that these interhemispheric interactions can be both inhibitory and facilitatory, depending on stimulus intensity [47, 48]. Here, our results seem to show that the stimulus (the motor task) had an inhibitory effect, since activation was more extensive in the absence of motor task. However, it might be interesting to investigate a possible facilitating effect of the motor task during MT on activation of the M1 contralateral to visual feedback by varying the intensity of the task.

To resume our results concerning M1, they can be explained by a facilitatory effect of VT or an inhibitory effect of MT. In both cases, the absence of a motor task in VT could lead to better stimulation of M1.

Finally, our results showed activation of the PMC contralateral to the visual feedback only during the VT task. This activation was limited to a single channel. This could correspond to activation of mirror neurons located in the ventral part of the PMC [49]. This system is particularly involved in action observation therapy [50]. As mentioned above, the VT used here could be considered as 1st-person AO, which leads to greater activation of mirror neurons than 3rd-person AO [51]. The activation found here could therefore be linked solely to movement observation (with no associated motor task) and would not be dependent on illusion perception, which is the basis of mirror therapy.

In summary, contralateral to the visual feedback, our results show a greater activation of PC during VT compared to MT and an activation of the motor cortex during MT and VT, but this activation was more extensive during VT. These results are mainly explained by the absence of a motor task during VT, which favours increased attention to the visual feedback (greater activation of PC) and possibly reduces interhemispheric inhibition mechanisms (larger activation of M1). Therefore, VT appears to optimise recruitment of the parietofrontal motor network compared with MT [52]. This network induces neuroplasticity after a brain lesion. Indeed, this network is more activated during a motor task in people after stroke than in healthy subjects [53]. Our results therefore suggest that VT may be clinically more effective than MT because of a greater stimulation of neuroplasticity, but this needs to be demonstrated in clinical studies of people after stroke. While the literature shows similar activation patterns between healthy subjects and stroke patients in the exploration of MT mechanisms [13, 14, 16, 17, 34], these mechanisms may be impacted by the location of the lesion. A study of 36 stroke patients showed that the clinical efficacy of MT was linked to the integrity of dorsal and ventral streams [54]. In view of the results of Brunetti et al. [19]. , , we can also assume that a lesion of the PC would also impact the clinical efficacy of MT. Thus, future studies in stroke patients, whether imaging or clinical, should consider results according to lesion location.

### Limitations and perspectives

This study has several limitations. First, the MT modalities differed from those used in clinical practice. Usually, individuals are asked to accompany the visualized movement by trying to move the impaired hand. This condition was not applicable to healthy individuals, as the intention would have resulted in a movement that would have masked the brain activation related to the visual feedback. Although this modality without movement intention can be applied to the patient, it does not appear to be optimal. A magnetoencephalography study in healthy individuals showed that contralateral M1 activation was greater when feedback was associated with the intention to perform the movement [15]. Another limitation concerns the task. We analysed a simple task (hand opening/closing), However, a study conducted in healthy individuals and people after stroke showed that MT-related brain activation was greater when the task was complex [55]. It would have been interesting to explore differences between simple and complex motor tasks.

Moreover, our study has some recruitment-related limitations. This study was carried out only on healthy subjects, whereas it investigates rehabilitation methods (i.e. MT and VT) used in the rehabilitation of stroke patients. While this was an important step, it will be necessary to evaluate these activation patterns and any clinical differences between the two techniques in stroke patients in future studies. In addition, we only recruited right-handed individuals, and the motor tasks were performed on the right side with left visual feedback. Therefore, our results cannot be extrapolated to the use of MT for the non-dominant side or to left-handed individuals. An electroencephalogram study of 13 healthy individuals found increased intracortical contralateral inhibition to movement and activation of mirror neurons [46]. However, the authors showed that the former effect was greater when participants moved their right (dominant) hand, and the latter was greater when the feedback was to the right (thus left-handed motor skills). To our knowledge, the specificity of left-handedness has not been evaluated in MT, but it is likely that different brain mechanisms are involved. Indeed, it has been shown that left-handed individuals have more bilateral activation patterns during the execution of motor tasks [56]. These different parameters should be considered in future studies.

Another limitation regarding the sample is that we only recruited young subjects, whereas some studies have shown that cortical activation patterns during motor task performance are different in older subjects. For example, one study showed that activation was more bilateral in older participants during a hand rehabilitation exercise using a multisensory glove [57]. Therefore, our results cannot be directly generalized to older adults or people after stroke, who are usually older than our study participants. Further studies in older subjects are thus warranted.

Finally, the fNIRS device did not allow us to cover the whole cortex. Therefore, we selected regions of interest (PC, PMC, and M1). However, other zones are activated during MT, such as the supplementary motor area, parietal and occipital cortices [34]. Unfortunately, this is a limitation of the fNIRS technique [12, 16]. In addition, some regions are not accessible by fNIRS, such as the cerebellum, which appears to be involved in MT [58].

## Conclusion

The results of this study reinforce the data in the literature concerning the mechanisms behind the effectiveness of mirror therapy and demonstrate the reliability of fNIRS for this type of exploration. Our results showed the involvement of a parieto-frontal network in which the precuneus appears to play a major role. This network seems to be more activated by VT than MT, which could be due in particular to the absence of a motor task. These results provide physiological data that could serve as a rationale for conducting clinical trials of activation patterns and efficacity in acute stage stroke patients.

## Data Availability

The data that support the findings of this study are available from the corresponding author on reasonable request.

## Abbreviations

MT: mirror therapy
VT: video therapy
M1: primary motor cortex
PMC: premotor cortex
PC: precuneus
HbO_2_: oxyhemoglobin
HbR: deoxyhemoglobin
fNIRS: functional near infrared spectroscopy
ROI: region of interest
DPF: differential path length factor
FDR: false discovery rate
AO: action observation
